# PlantSimLab - a modeling and simulation web tool for plant biologists

**DOI:** 10.1186/s12859-019-3094-9

**Published:** 2019-10-21

**Authors:** S. Ha, E. Dimitrova, S. Hoops, D. Altarawy, M. Ansariola, D. Deb, J. Glazebrook, R. Hillmer, H. Shahin, F. Katagiri, J. McDowell, M. Megraw, J. Setubal, B. M. Tyler, R. Laubenbacher

**Affiliations:** 1 Department of Computer and Information Sciences, Virginia Military Institute, Lexington, VA USA; 2 School of Mathematical and Statistical Sciences, Clemson University, Clemson, SC USA; 3 Biocomplexity Institute of Virginia Tech, Blacksburg, VA USA; 4 Virginia Tech, Blacksburg, VA USA; 5 Celgene, San Francisco, CA USA; 6 Department of Natural Sciences, Mercy College, Dobbs Ferry, NY USA; 7 College of Biological Sciences, University of Minnesota, St. Paul, MN USA; 8 Mendel Biological Solutions, San Franciso, CA USA; 9 Department of Plant Pathology, Physiology, and Weed Science, Virginia Tech, Blacksburg, VA USA; 10 Department of Botany and Plant Pathology, Oregon State University, Corvallis, OR USA; 11 Biochemistry Department, University of Sao Paolo, Sao Paolo, Brazil; 12 The Jackson Laboratory for Genomic Medicine, Farmington, CT USA; 13 Center for Genome Research and Biocomputing, Oregon State University, Corvallis, OR USA; 14 Center for Quantitative Medicine, School of Medicine, University of Connecticut, Hartford, USA

**Keywords:** Mathematical model, Biological network, Dynamic network model, Plant biology, Modeling software

## Abstract

**Background:**

At the molecular level, nonlinear networks of heterogeneous molecules control many biological processes, so that systems biology provides a valuable approach in this field, building on the integration of experimental biology with mathematical modeling. One of the biggest challenges to making this integration a reality is that many life scientists do not possess the mathematical expertise needed to build and manipulate mathematical models well enough to use them as tools for hypothesis generation. Available modeling software packages often assume some modeling expertise. There is a need for software tools that are easy to use and intuitive for experimentalists.

**Results:**

This paper introduces PlantSimLab, a web-based application developed to allow plant biologists to construct dynamic mathematical models of molecular networks, interrogate them in a manner similar to what is done in the laboratory, and use them as a tool for biological hypothesis generation. It is designed to be used by experimentalists, without direct assistance from mathematical modelers.

**Conclusions:**

Mathematical modeling techniques are a useful tool for analyzing complex biological systems, and there is a need for accessible, efficient analysis tools within the biological community. PlantSimLab enables users to build, validate, and use intuitive qualitative dynamic computer models, with a graphical user interface that does not require mathematical modeling expertise. It makes analysis of complex models accessible to a larger community, as it is platform-independent and does not require extensive mathematical expertise.

## Background

### Motivation

“*Like most mathematicians, he takes the hopeful biologist to the edge of a pond, points out that a good swim will help his work, and then pushes him in and leaves him to drown*.” (C. Elton, in a 1935 review of work by A. Lotka) [1]. The modern biologist might well have the same reaction when confronted with many of today’s mathematical models and software tools. The ideal approach to (plant) systems biology that avoids this problem might be interdisciplinary research teams integrating biology and computation, with significant overlap in skill sets. This is unfortunately not the every-day reality in the short or medium term. A widespread adoption of tools that bring computation and systems “thinking” to the study of dynamic molecular pathways requires that a biologist use them without access to a modeler and without acquisition of advanced mathematical skills. PlantSimLab, the software package described in this paper, represents an attempt to address this reality. We aimed to build an intuitive tool with a shallow learning curve and some basic utilities, a “flip phone” of modeling tools for the uninitiated, rather than a “smart phone.”

Systems biology is a useful approach to plant biology, and biology in general, at several different scales (see, e.g., [2]). Focusing on systems-level dynamic phenomena naturally relies on extensive use of mathematical models. If the interest is in elucidating network topology, then typically tools from graph theory are used to study connectivity features. If the interest is in studying dynamic effects, then one needs to build and analyze dynamic computer models of networks. In both cases, two approaches are used: the so-called “bottom-up” approach, which builds a model of the network from available information about its components, and the “top-down” approach, which extracts network links from experimental, typically high-dimensional, data. Ideally, both approaches are used in combination. The software tool described here in its current form is entirely bottom-up. It provides a graphical user interface that allows the construction of dynamic models of networks, their simulation, and the basic experimental capability of knocking out a node. The fundamental hypothesis underlying PlantSimLab is that, with the right type of modeling paradigm and the right interface, biologists can themselves construct and manipulate useful mathematical models for hypothesis generation, without having expert knowledge or a background in mathematics or modeling. The goal is to provide biologists with an easily usable, virtual laboratory tool to integrate available information and data for the purpose of hypothesis generation. These constraints require a mathematically simple way of constructing models and interpreting model output, making modeling frameworks such as differential equations less well-suited. We have chosen the modeling paradigm of time- and state-discrete dynamical systems, which are essentially generalized Boolean networks. They have been used successfully to capture a wide range of molecular networks in recent years; see, e.g., [3–6].

### Existing software packages

There are a number of excellent software platforms available for modeling using Boolean networks and their generalizations. The Cell Collective [7] is an interactive web tool built with a special emphasis on the collaboration of distributed teams to build large Boolean models of molecular networks; it has many features and an extensive library of models. The popular web tool GinSim [8] provides a graphical user interface to build and analyze so-called logical models, which can be viewed as generalized Boolean networks with additional features. The R package BoolNet [9] is a very convenient approach to building and simulating stochastic Boolean models, with randomly varying update schemes for the variables. Several other more recent platforms have been developed; see, e.g., [6, 10–13] for a partial list. Some of these can deal only with Boolean networks, such as BoolNet, while others can handle multi-state models, such as GinSim. They have in common, to a greater or lesser extent, the assumption that the user has some modeling experience or is willing to undertake a relatively steep learning curve. All of them have a wide variety of features, whether it is the accommodation of different modeling frameworks, the ability to infer dynamic models from data, or features that allow sharing and distributed model construction.

### Our contribution

The modeling platform described here differs from these platforms primarily through its relative simplicity, and ubiquitous default settings that significantly shorten the path to a working model. The user can choose any (finite) number of states for any of the nodes, with state labels chosen from a predetermined menu (with the option to customize). The user then chooses edges between nodes from a set of default choices (e.g., activate, inhibit, custom). For activating or inhibiting edges, an autofilled transition table appears (which can be customized, if desired) that specifies the action of the edge, taking into account the respective numbers of states for source and target nodes and their labels. We have chosen to use basic transition tables to describe the logical rules for the way each node takes up and integrates its different regulatory inputs. The advantage is that, in essence, each row in a transition table represents a biological statement, such as “when *A* is high, *B* is low, and *C* is low at time *t*, then *C* (which is regulated by both) transitions to being medium at time *t*+1,” corresponding to the row [high low low | medium], which, for mathematical purposes gets translated into [2 0 0 | 1]. Thus, model construction is simplified as much as possible. Model analysis in the current version is limited essentially to computing the different steady states the model is capable of, corresponding to the different phenotypes exhibited by the system to be modeled. Basic “experiments” that can be performed with the model include “knock-out” of one or more nodes and the edges connected to the knocked-out node(s), and the ability to observe the resulting changes in system behavior. In our experience, this “bare-bones” approach is effective in providing quick model construction and a check on the consistency of the assumptions underlying the model. This is the first step in using the model for hypothesis discovery. The other existing modeling platforms described above may then serve as an “upgrade” for biologists with more extensive modeling expertise.

## Implementation

The purpose of the software is to let the user construct a dynamic model of a molecular (or other) network from biological knowledge, and allow a basic exploration of model dynamics as well as the effect of certain perturbations. In order to simplify model construction, a number of default settings are used that can subsequently be customized by the user, prioritizing simplicity and speed. The user first constructs a network in the form of a directed graph using a simple user interface, which indicates the causal dependencies of the network nodes. Our modeling framework of choice is that of dynamic models that are time-discrete, i.e., variables are updated in discrete time steps, and state-discrete, i.e., each variable can take on a finite number of possible states (currently up to five), and this number can vary across variables. The result is a finite (but conceivably large) space of possible system states (given in the form of a directed graph, with directed edges indicating state transitions). Each network node has attached to it a function that takes as input the states of all of the nodes from which there is an incoming arrow, and provides as output the “next” state of the node. As a special case, each node could take on exactly two values, resulting in a Boolean network. Such a function can be specified in a number of ways, for instance through a Boolean function in the case of binary inputs. We have chosen the most simple and intuitive description, through the specification of a transition table that specifies the output for each possible input vector of states. Such a table is automatically generated by default, integrating the different inputs in an additive fashion. The table can subsequently be customized, for instance, to use synergistic action instead of additive. Each row of such a table can be interpreted as a biological statement, e.g., “If A is high, B is low, and C is high at time *t*, then C will become low at the next time step,” representing the row [1 0 1 | 0] in the Boolean case. Thus, there is no need to learn any mathematical formalism to specify functions. The user is able to carry out basic computational “experiments,” namely to knock out network nodes and the arrows/interactions connected to those nodes. Finally, the user can analyze the model by computing all steady states, typically corresponding to different cellular phenotypes and attractor basins, corresponding to the relative likelihood of that phenotype.

The fundamental algorithm underlying all these calculations exhaustively enumerates all possible state transitions from the transition table. This is done as follows, using the binary case as an illustration. For a model with *n* nodes, the 2^*n*^ possible network states are arranged alphabetically. The algorithm takes the first state, **x** = (0, 0, …, 0) as input and computes the “next” state, **y**, using the transition table. The new state now becomes the input to the algorithm, which first checks whether **x** = **y**. If yes, it picks the next state in the transition table that has not be used yet. If no, then it computes the next state **z**, using the row of the transition table corresponding to **y**. It then checks whether **z** has appeared earlier in the process. If so, a cycle has been found and the algorithm moves on to the next state not yet used as input. The algorithm ends when all 2^*n*^ states have been used as input. Several other possible algorithms could be used for the same purpose.

### User Interface

The graphical user interface (GUI) guides the user interactively through the modeling and analysis steps. Four arrow-shaped tabs are displayed along the top of the canvas rectangle in the natural order of constructing a model, setting up and carrying out computational experiments, and analyzing the results. The transitions between the different modes can be done automatically according to the functional process of modeling activities or manually by clicking any arrow tab to open a functionality. The currently open mode is always indicated by a green color highlight on its tab, so the user knows which mode is currently selected. We now describe these four modes in more detail. We will use the following small generic model as a running example, which can also be found on the PlantSimLab website:

*A* → *C* ← *B.*

where *A* has two states (0, 1), *B* has three states (0, 1, 2), and *C* has four states (0, 1, 2, 3). Here, *A* has an activating influence on *C*, and *B* has an inhibiting influence.

#### Model editor

This tab provides a canvas drawing area and a suite of graphical model-editing tools for the user to draw a network model that is a graph-theoretic representation of the molecular network of interest. The user can create a node by clicking on the node icon in the toolbar and then clicking the location to place the node on the canvas. Then the user chooses the number of states for the node with state labels chosen from a predetermined menu or customized. Internally, the states get converted into numerical values, beginning with “0” for the first state in the table, up to “*n*-1,” where *n* is the number of states (currently limited to five).

To create an edge, the user can click on an edge icon (with choices including “activate,” “inhibit”, or “unspecified”) in the toolbar, and then click the input and the target node successively. For an “activate” edge, a state transition table is created that captures the effect of states of the input node on the states of the output node. This table can then be modified by the user, similar to the table for an edge whose nature is unspecified. If a node has several input edges, their effects are combined into a comprehensive transition table called “Big State Transition Table” (BSTT), where the different inputs are integrated using an “additive” rule by default. That is, if a node receives two or more inputs, then the input values are added (e.g., if the edges are activating), respectively subtracted (e.g., if one or more nodes are inhibiting), depending on the edge tables at each time step. We use the arithmetic convention that the resulting number cannot be lower than 0 or larger than *n*-1. Again, the user can customize this default choice. Several editing features simplify the management and editing of large tables.

### Calculation of state transitions

**Fig. 1:**
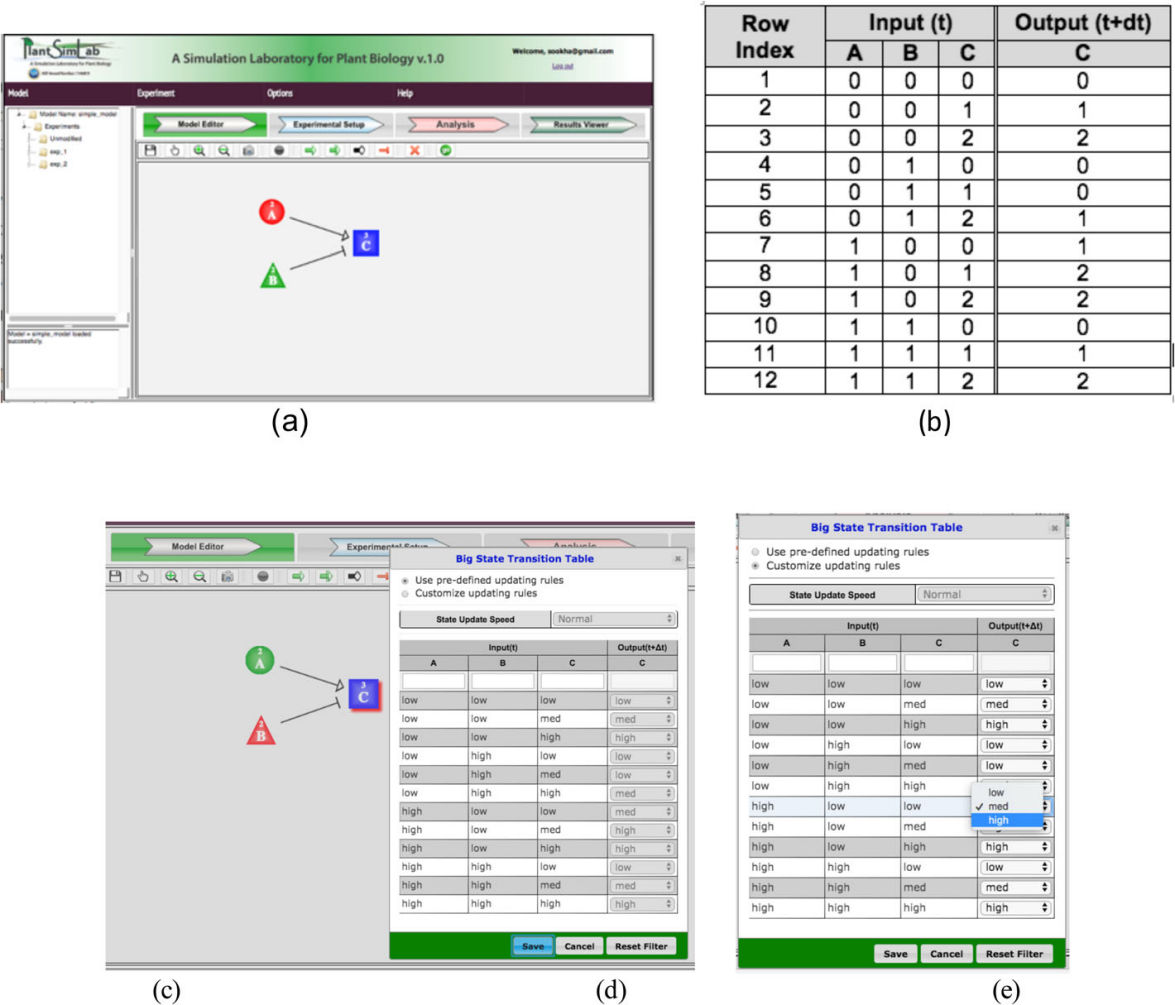
The wiring diagram of a simple network model containing three nodes (**a**). A table to show the calculation of all possible state transitions for a target node *C* in the network model (**b**). Double clicking on a node on the *Model Editor* canvas highlights the node in the back and opens up the Big State Transition Table (BSTT) for the node (**c**). The user can use the predetermined choice (**d**), or change it by selecting a desired state from the dropdown box in the row (**e**)

To show how PlantSimLab calculates the predetermined state transitions for a node, we created a very simple network model having only three nodes named “*A*” (2 states), “*B*” (2 states), and “*C*” (3 states), where node *A* activates node *C,* and node *B* inhibits node *C* (Fig. 1a)). The table in Fig. 1b is the big state transition table (BSTT) for node *C*. It displays all possible combinations of the input node states in the current time cycle and their corresponding target node states in the next time cycle. Based on additive rules, PlantSimLab calculates the state of the target node *C*_*t*_ (*C* at time *t*) in the next time cycle, represented as *C*_*t* + *dt*_, for any possible combinations of the input nodes *A*, *B*, and *C* at time *t* as follows. The table entries in Fig. 1b for which *B* is equal to 0 give the effect of *A* alone on *C*. Likewise, the entries for which *A* is equal to 0 give the effect of *B* alone. The cumulative effect is assumed to be additive, in the sense that the right-hand column of the table is obtained as follows:

$$ {C}_{t+1}={A}_t-{B}_t+{C}_{t,} $$

subject to the constraint that the value is equal to 0, if *A*_*t*_-*B*_*t*_ + *C*_*t*_ *<* 0 (integer arithmetic), and equal to 2 if *A*_*t*_-*B*_*t*_ + *C*_*t*_ > 2.

Currently, PlantSimLab allows up to 5 states for a node, rendered as 0, 1, …, 4. The exhaustive enumeration of all state transitions is calculated using the same additive rule with adjustment for the lowest and the highest possible state values in this way. The BSTT opens automatically when the node is double-clicked (Fig. 1c). The user can use the default choice (Fig. 1d), which completes the right-hand column in Fig. 1b using the tables for the arrows from *A* and *B*. Or the user can change it by selecting a desired state from the dropdown box in the appropriate row of the BSTT (Fig. 1e).

A PlantSimLab network model can be thought of as a wiring diagram, with metadata attached, and the content of the canvas can be saved as an image file on the user’s local machine for inclusion in publications or presentations. When a network model is created in *Model Editor*, the user can explore the network dynamics of the unchanged model by running the Dynamical Network Analysis algorithm with a click on a short-cut button “go” provided in the *Model Editor* toolbox. In particular, the user can observe select time courses of model states beginning with initial states of interest, as explained below.

### Experimental setup

This tab provides a canvas drawing space and a menu of experiments, which currently consists of the possibility to simulate the knock-out of one or more nodes.

**Fig. 2:**
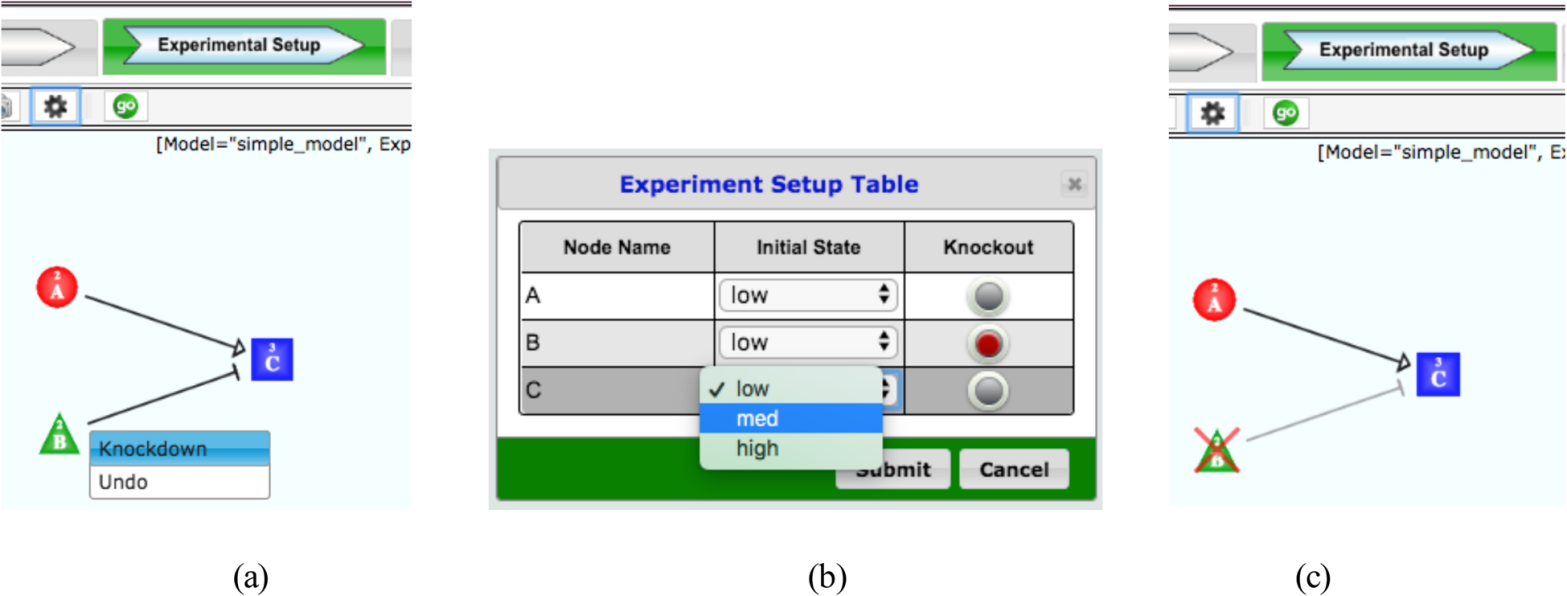
For an experiment, the user can perturb a model by knocking out nodes and the associated edges. The user can do this on a single node using a context menu popping up after right-clicking on a node to knock out or undo (**a**), or using the Experimental Setup Table. The initial state of the node can be set to the desired state in this tab using Experiment Setup Table (**b**). A knocked-out node has a X mark in red through it (**c**)

The default setting for a knocked-out node is constant equal to the node state corresponding to “0” for all simulation time steps. This can be done for several nodes simultaneously. The user can customize this feature and set the state of a “perturbed” node constant equal to any chosen state. In this way, one can also simulate knock-down and overexpression of one node or a combination of these for more nodes simultaneously. Knocking out a node or reversing a knockout can be done on a single node using a context menu popped up at the right-click on a node to knock out or undo (Fig. 2a), or on multiple nodes at once using the Experimental Setup Table (Fig. 2b), which provides a knockout button for each node so the user can select the multiple nodes to knock out or undo the knockout all at once.

### Model analysis

From a given initial state, the network can evolve to a steady state or a collection of states through which the network cycles, exhibiting oscillatory behavior. A given network might be capable of several different such behaviors, depending on the chosen initial state. The software computes all such steady states and collections of oscillatory nodes, collectively referred to as attractors. The terminology refers to the feature that once the network reaches either a steady state or a collection of states that is oscillatory, it remains there. The basin of attraction for a given attractor simply refers to the collection of states for which the network evolves to that attractor. The size of the basin of attraction, that is, the number of states in it, gives an indication of how likely the attractor is to occur. Thus, model analysis provides a comprehensive view of the dynamic range the network is capable of. In many cases, interest is focused on a particular initial state or small collection of initial states. The user can then simply focus on the time evolution of the network from these states and ignore all other information.

### Results viewer

**Fig. 3:**
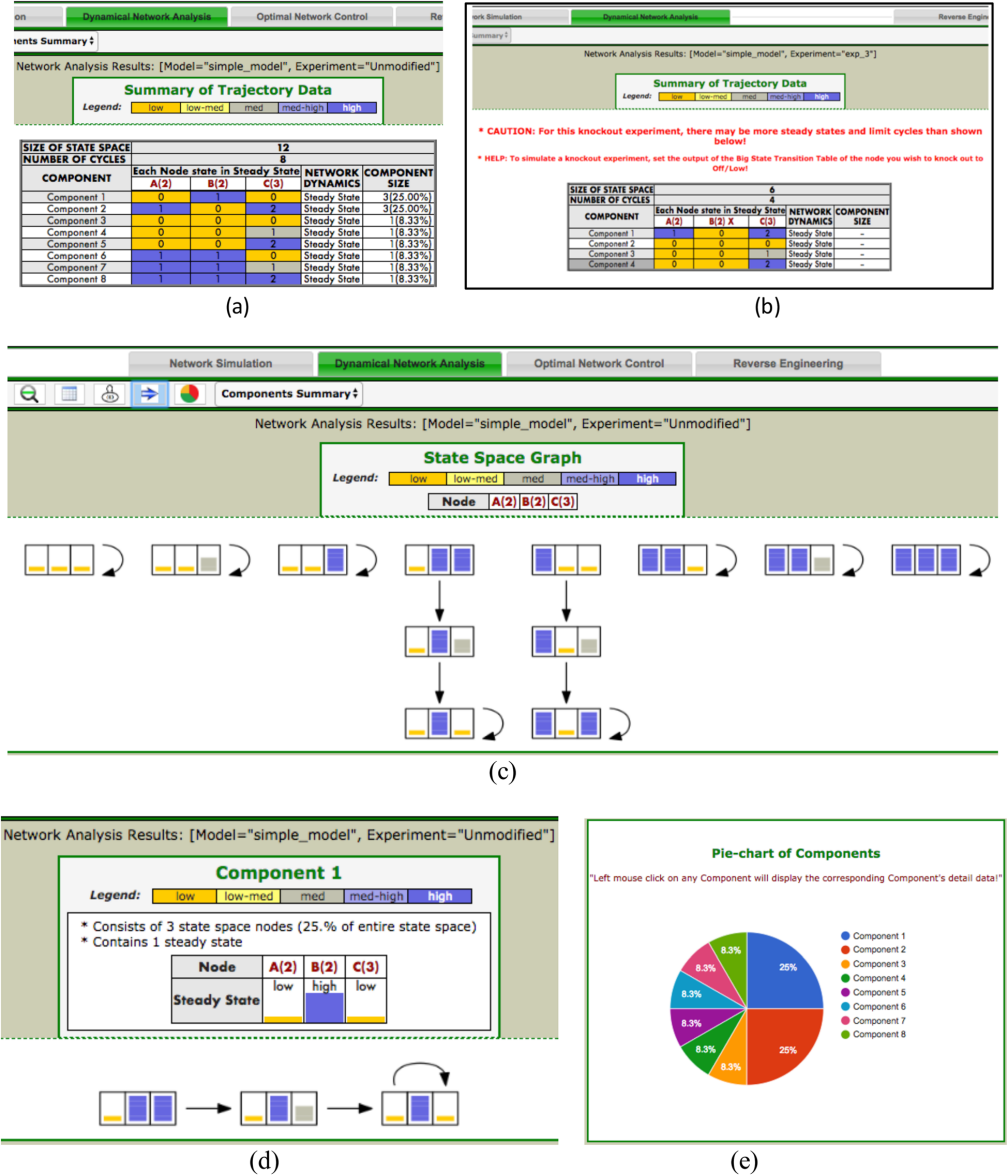
The summary Table (ST) displays all attractors and the attractor basin in the system for the running example model with three nodes introduced above. The HTML table uses a heat-map style color scheme to display the steady states of nodes. The ST on the left is generated for an unchanged model (**a**) and the ST on the right is for a perturbed model (**b**). For all perturbed models, PlantSimLab displays a CAUTION message to inform of the potential existence of other steady states or limit cycles than the displayed (**b**) (see below for an explanation). The entire state space graph is drawn using different colors and box sizes to make the state space graph more visually intuitive and informative (**c**). A subset of the state space (**d**) can be also drawn for further study of the simulation of a network component selected with a click on a row corresponding to a particular component row in the ST, on a component piece in the pie-chart (**e**), or on a component entry from the Component Summary drop-down box provided in the Results Viewer toolbox

This tab is designed to display the network analysis results using various HTML forms, styles, and devices to deliver the output information in an intuitive and easy to understand format. The summary Table (ST) shows all attractors of the dynamic network and their basins of attraction. The magnitudes of the node states in the attractor are presented by their numerical values as well as in a heat-map-style color scheme to enable a quick grasp of the information about the attractor. The relative contribution of each attractor basin to the entire state space is given as a percentage. A pie-chart combining all components into a compact view also enables a quick visual gauge of the relative contribution of each attractor. Clicking on a particular row in the ST or a pie segment from the pie-chart opens up a separate window and displays the detailed information about the selected subset of the state space, including the state space graph of the subset. To create a more intuitive and easy to understand space state graph, we used HTML style bar size and color coding for presenting the state of a node. A cell for a low state node in the graph is filled with a small bar in yellow, for a high state node with a full size bar in purple, and for a medium state node with a half size bar in gray (Fig. 3d). For better visibility, the user can control the direction of the state space graph display horizontally or vertically using toggle buttons. The user will notice that the state transition of any knock-out node remains constant equal to the state corresponding to 0. The ST can be conveniently saved into an Excel file on the user’s local machine.

#### Known issues/limitations

Due to space and computation time limitations, PlantSimLab does not draw the state space graph if the size of the state space exceeds 2600 nodes. Furthermore, the current algorithm used for dynamical network analysis was not built to handle models with knocked-out nodes; rather, it automatically enumerates all possible state transitions of all nodes by default. This limits the functionality of the network perturbation feature. To partially resolve the issue, PlantSimLab currently excludes the output display of those steady states or limit cycles where the knocked-out nodes are not in their lowest state. As a result, for a perturbed network only some of the steady states and none of the limit cycles are generated. The user is notified about this through a warning message whenever a perturbed network analysis is performed. The message advises the user to instead simulate a node knockout by setting the node state to low/off in the BSTT if complete information on the steady states is desired.

#### YouTube tutorials

We created three YouTube tutorial videos to provide instructions on how to create, perturb, and analyze a network model, and view the network analysis results in PlantSimLab for hypothesis generation. Tutorial #1 shows how to create nodes and edges, and configure them interactively using special tables for building a graphical network model in the *Model Editor tab*. Tutorial #2 shows how to perturb a model by knocking out nodes in the *Experimental Setup tab*. Tutorial #3 explains how to visually inspect the dynamical network analysis results in many different forms in the *Results Viewer* tab. All instructions in these tutorials are easy to follow and it takes only 5 to 11 min for each video. The three PlantSimLab YouTube videos are available through the following links:

PlantSimLab Tutorial #1 - Model Building [14].

PlantSimLab Tutorial #2 – Experimental Setup [15].

PlantSimLab Tutorial #3 – Network Analysis Results Viewer [16].

## Development

### Software components

We developed PlantSimLab as a client-server-based web application running on Apache2 on a dedicated server and supporting most modern web browsers on any platform. The user interface on the client was developed using HTML with significant components of JavaScript and AJAX to enhance the user experience. On the server side we used PHP scripting and an open source MySQL database for model repository management. The use of Google sign-in authentication for the user login promotes user convenience and reduces the burden of user profile management for the software.

To create an intuitive, well-designed, and frustration-free user interface, we applied design principles similar to Shneiderman’s ‘eight golden rules of interface design’ [17].

#### A use case

To demonstrate the use of PlantSimLab, we implemented and analyzed the model from Espinosa-Soto et al. [18]. This paper follows several other investigations into the gene networks driving cell fate determination in the model organism *Arabidopsis thaliana.* The authors focus on the question of robustness of morphological pattern development, in particular floral organ cell fate determination. Experimental studies led to the development of the ABC combinatorial model of gene expression states that predicts the identity of floral organ primordia, which has guided many experimental studies. The model presented in [18] is based on a more complete understanding of the genetic components and interactions involved, resulting in model steady states that are coherent with experimental data. The main finding in [18] is that all possible initial conditions converge to a few steady states that match experimental observations. Thus, the network provides a dynamic explanation of the ABC model and shows that precise signaling pathways are not required to restrain cell types to those found in *Arabidopsis,* but these are rather determined by the overall gene network dynamics. The cell types recovered depend on the network architecture rather than on specific interaction parameters. Finally, these results support the hypothesis that such a network constitutes a developmental module, and hence provide a possible explanation for the overall conservation of the ABC model and overall floral plan among angiosperms. We now illustrate how this model can be built and analyzed in PlantSimLab. Once the logical rules are extracted from the paper, the model can be built in the software within a matter of hours.

**Fig. 4:**
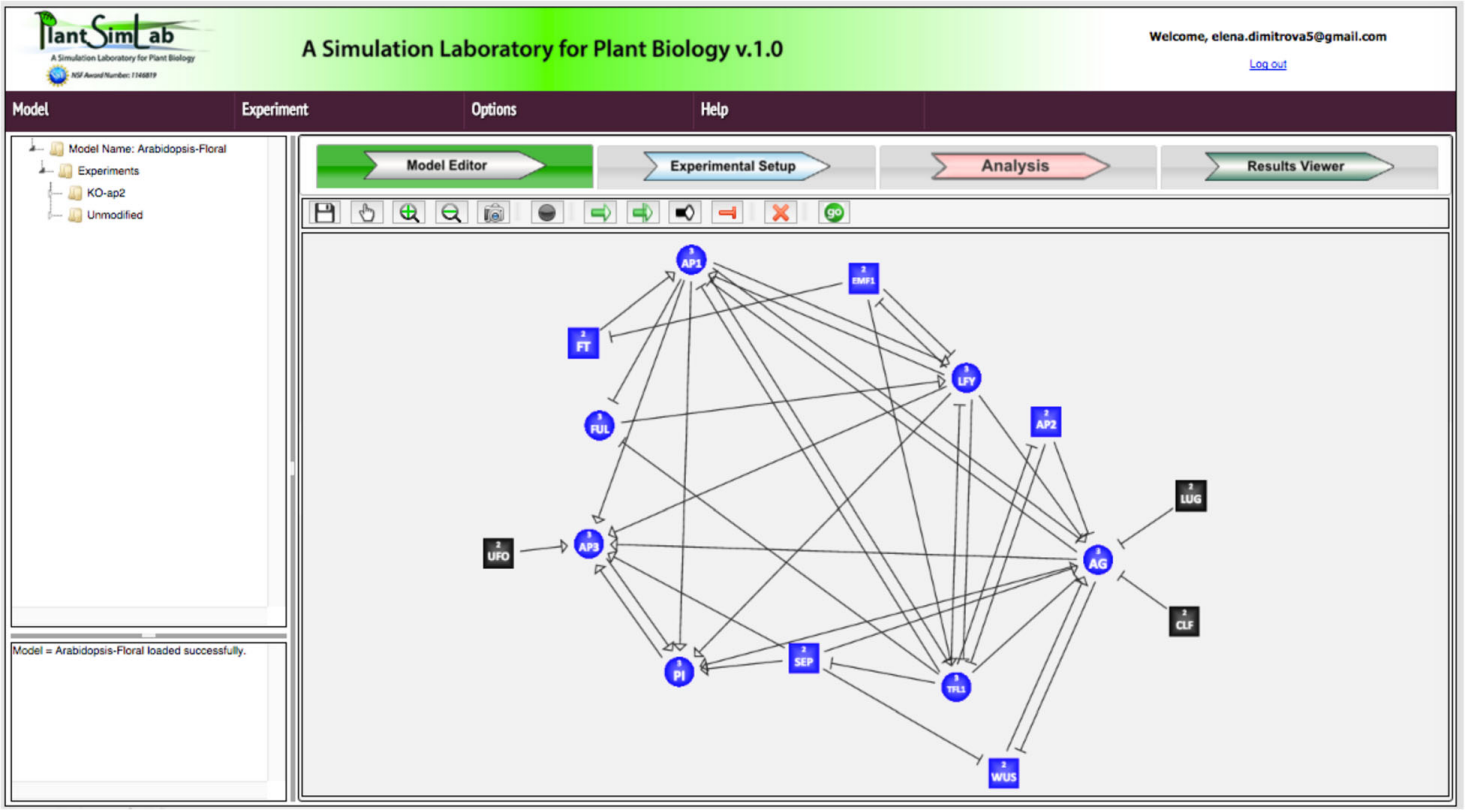
The software components (**a**) and the software workflow (**b**) of PlantSimLab, highlighting the steps for modeling, analysis, and use. PlantSimLab communicates with the model database repository to load and save user models. To perform network analysis, PlantSimLab runs a Dynamical Network Analysis algorithm, a locally installed application on the server (**b**)

The model consists of 15 nodes, eight of which can assume two states, e.g. ON/OFF (FT, EMF1, SEP, AP2, WUS, UFO, CLF, and Lug) and seven can assume three states, e.g. LOW/MEDUIM/HIGH (LFY, AP1, FUL, TFL1, AG, AP3, and PI). The wiring diagram of the model generated by PlantSimLab is presented in Fig. 4**,** and is identical to Fig. 5 in [18]. In [18], the logical rules for each node are provided in table form for each node, very similar to PlantSimLab’s transition tables. We implemented in PlantSimLab the proposed wild-type network and the loss-of-function *ap2* mutant. The simulation of the wild-type network generated a total of 40 steady states and no other cycles (Fig. 6). Among them were the 10 steady states recovered in [18], based on the 139,968 initial conditions considered there (the total number of possible initial conditions is 559,872). The simulation of the *ap2* mutant network returned 28 steady states and no other cycles (Fig. 7), with the seven steady states simulated in [18] present among them. We note that both simulations considered all possible initial conditions and, as a result, were able to generate all network steady states, with some of the additional ones having relatively large basins of attraction (up to 22% of all states). It took PlantSimLab only seconds to complete the simulations, and the additional steady states that were discovered may provide valuable additional information about other possible phenotypes of the cells considered.

**Fig. 5:**
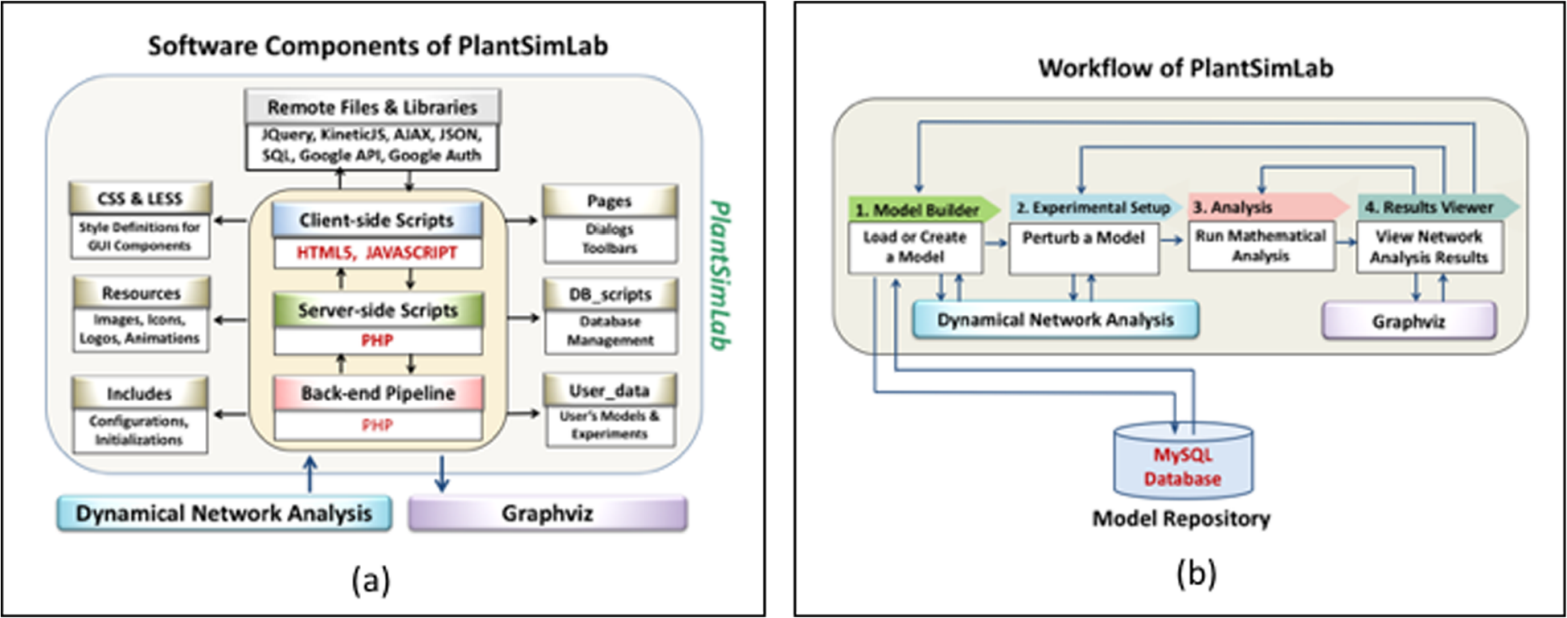
Wiring diagram of the network, identical to Fig. 4 in [18]

**Fig. 6:**
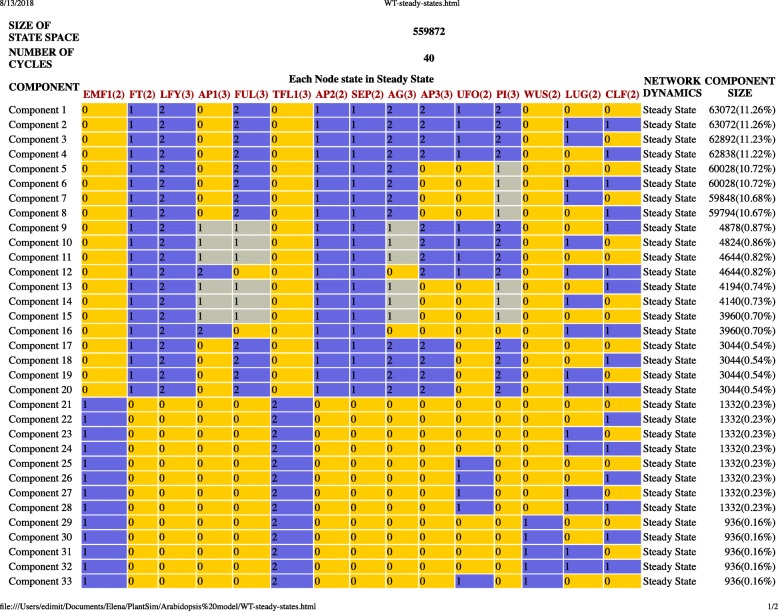
The list of steady states and component sizes from the wild-type network simulation

**Fig. 7:**
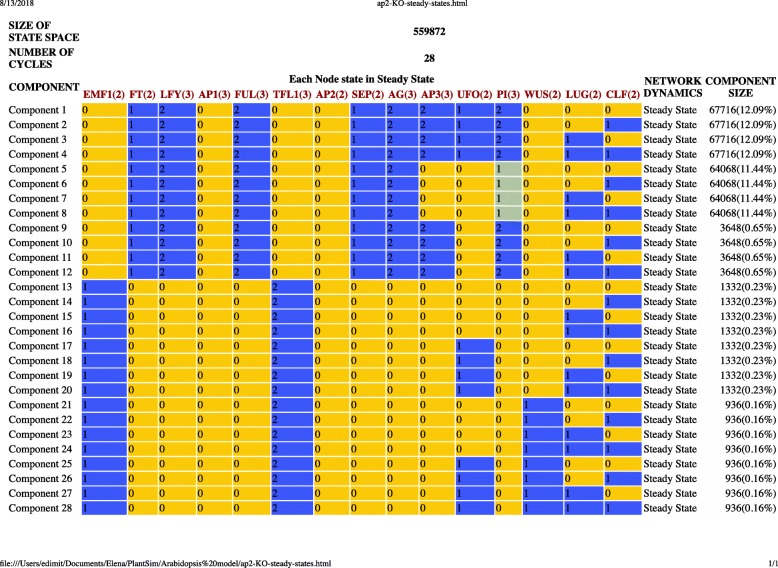
The list of steady states and component sizes from the *ap2* knock-out network simulation

While this software package was developed with applications to plant biology in mind, the tool is in fact quite generic and can be applied in a variety of settings. To illustrate this and to provide the user with further examples to explore the software, we have included three additional models in the model library**.** One is a model of the *lac operon*, one of the earliest examples of a gene regulatory network, taken from [19]. The other two models capture some key features of the immune response to vascular injury, published in [20]. One of the models captures signaling events in a macrophage recruited to the injured tissue from the circulation, the other models tissue-level events of a population of macrophages. The three models together show the versatility of PlantSimLab for purposes other than plant biology.

## Conclusions

In this work, we present the web-application tool PlantSimLab, a simple, intuitive software tool for creating dynamic network models, performing experimental simulations, and visualizing network analysis results using a variety of formats and dynamic layouts. It provides a computational laboratory for biological scientists to generate novel experimental hypotheses. It is designed to be usable after completing a brief online interactive tutorial that explains the basic input, output, and functionalities of the software. It was our goal to make all features of PlantSimLab’s interface as intuitive and self-explanatory as possible for life science researchers. Future development of the software tool includes a simple natural language parser that allows users to provide English language sentences with prescribed syntax, such as “*A* [interacts with] *B*,” which are then automatically translated into network components. The YouTube tutorials will be extended to cover more advanced technical topics.

We add some miscellaneous comments here. One of the features available in other modeling software tools, such as *GinSim*, mentioned earlier, is the capability of model checking [21–23]. Among other features, this allows the user to specify constraints the model is required to satisfy, which are then automatically verified. While this feature is very useful for model building, we have opted not to include it in this initial version of the software, which, as explained in the introduction, is focused on implementing the most intuitive and simple functionalities for model building. We plan to include it in future releases, however. As a second comment, we want to emphasize that the software allows the user to specify very general regulatory functions, in addition to the additive mechanisms used for the default settings. The user can specify as much or as little of the transition table for a given edge or node as desired, with the remainder completed as a default setting. In particular, the user can specify the **entire** transition table without constraints, so that arbitrary functions can be used.

## Availability and requirements

Project Name: PlantSimLab.

Project home page: http://app.plantsimlab.org

Operating System(s): Platform independent (Linux, Windows, MacOS).

Programming languages: HTML5, JavaScript, PHP, CCS/LESS, SQL.

Any restriction to use by non-academics: None.

Licenses: None.

Other requirements: A Google email account to sign in.

## Data Availability

No data have been used in this project. The models used to illustrate PlantSimLab were built based only on information available in the publications cited in the text.

## Abbreviations

AJAX: Asynchronous Java Script and XML
BSTT: Big State Transition Table
GUI: Graphical User Interface
HTML: Hypertext Markup Language
MySQL: Open source relational database management system
PHP: Hypertext Processor
ST: Summary Table
